# Long lasting near-obstruction stenosis of mesencephalic aqueduct without development of hydrocephalus – case report

**DOI:** 10.3325/cmj.2014.55.394

**Published:** 2014-08

**Authors:** Milan Radoš, Darko Orešković, Marko Radoš, Ivana Jurjević, Marijan Klarica

**Affiliations:** 1 Croatian Institute for Brain Research, University of Zagreb School of Medicine, Zagreb, Croatia; 2 Department of Molecular Biology, Ruđer Bošković Institute, Zagreb, Croatia; 3 Department of Clinical and Interventional Radiology, Clinical Hospital Center Zagreb, University of Zagreb School of Medicine, Zagreb, Croatia; 4 Department of Neurology, Clinical Hospital Center Zagreb, University of Zagreb School of Medicine, Zagreb, Croatia; 5 Department of Pharmacology, University of Zagreb School of Medicine, Zagreb, Croatia

## Abstract

The aim of this study is to present the five-year longitudinal magnetic resonance imaging (MRI) follow up of a patient with incidental finding of near-obstruction stenosis of the aqueduct of Sylvius due to a large pineal cyst. The patient was scanned 3 times on a 3T MR device using a set of standard structural sequences supplemented with high-resolution constructive interference of steady state (CISS) T2 sequence for precise delineation of the aqueduct of Sylvius and cardiac-gated phase-contrast sequences for the analysis of cerebrospinal fluid (CSF) movement. On all MR scans, the size of the pineal cyst and severity of near-obstruction aqueductal stenosis did not show any morphological changes. There was no significant ventricular enlargement although structural CISS sequence showed a near-obstruction stenosis and cardiac-gated phase-contrast sequences did not detect CSF movement through the aqueduct of Sylvius. Our findings are contradictory to the classic hypothesis of CSF physiology based on secretion, circulation, and absorption of CSF, which states that the impairment of CSF circulation through the aqueduct of Sylvius inevitably leads to a hypertensive hydrocephalus development involving the third and the lateral ventricle. Our research group previously proposed a new hypothesis of CSF physiology, which offers more suitable explanation for such clinical cases.

Clinical practice has shown that an aqueduct of Sylvius obstruction or severe stenosis (tumoral compression, intrinsic non-tumoral pathology) could lead to a development of the triventricular hypertensive hydrocephalus (1). According to the classic hypothesis of CSF physiology, CSF is secreted inside the brain ventricles and flows unidirectionally along the subarachnoid space to be absorbed into the dural venous sinuses or into perineural lymphatic system (2,3). Therefore, a blockade of CSF flow from the third to the fourth lateral ventricle at the aqueduct of Sylvius level inevitably leads to the triventricular hypertensive hydrocephalus development. It is assumed that CSF is formed by an active secretion mainly by the choroid plexuses against hydrostatic pressure inside the CSF system, so any blockade between the site of secretion and the site of absorption will lead to CSF accumulation, CSF system enlargement, and subsequently to the development of hydrocephalus proximal to the obstruction site (1).

The aqueduct of Sylvius is a narrow canal inside the mesencephalon that connects the third and the fourth lateral ventricle, thus, according to the classic hypothesis, its passability is of key importance for normal CSF physiology (2,3). An aqueduct of Sylvius stenosis sometimes leads to the compensatory changes of the CSF system that are known as “arrested hydrocephalus” (4). The compensatory mechanisms of this state are still unclear. We present a patient with long lasting near-obstruction stenosis without detectable CSF movement through the aqueduct of Sylvius, which has some similarities with the “arrested hydrocephalus” condition. Despite the functional blockade of CSF movement, no significant ventricular enlargement was observed during the follow up period of 5 years. Such findings are contradictory to the classic hypothesis of CSF hydrodynamics and classic circulatory hypothesis of hydrocephalus development. However, the new hypothesis of CSF physiology proposed by our team (Bulat, Orešković and Klarica hypothesis) (5-7) states that stenosis/blockade of aqueduct of Sylvius could favor but does not necessarily lead to the development of hydrocephalus (6,8). In this case report, we discuss pathophysiological mechanisms of hydrocephalus development from the perspective of both classic and new microcirculatory hypothesis of CSF physiology (5-7).

## The patient

The patient presented in this case is a 40-year old woman with 25-year-long history of epilepsy, which is well controlled by 1 mg of clonazepam (the last seizure occurred before 15 years). She does not have a history of any other clinical symptoms (headache, nausea, vomiting, ataxia, dementia, etc). Structural brain magnetic resonance imaging (MRI) performed as a routine diagnostic procedure for epilepsy revealed a large pineal cyst that compressed the quadrigeminal plate. Control follow up MRI exams were performed four and five years after the first exam. The patient’s informed consent was obtained before every MR exam. All MR exams were performed at the Policlinic Neuron at the Croatian Institute for Brain Research in 2009-2014 period.

All MRI exams were performed on a 3T MR scanner (Magnetom TrioTim, Siemens, Erlangen, Germany) using 12-channels head-coil. At the first MRI exam standard MR sequences for epileptic patients were used and the aqueduct of Sylvius was analyzed on sagittal T1 sequence (TE/TR = 440/2.5 ms; matrix = 256 × 256; FOV = 22 × 22 cm; voxel size = 1 × 1 × 4 mm). At the second MRI exam, standard protocol was supplemented with a high-resolution magnetization-prepared rapid acquisition with gradient echo (MPRAGE) sequence (TE/TR = 1900/2.5 ms; matrix = 256 × 256; FOV = 25 × 25 cm; voxel size 1 × 1 × 1 mm). At the third MRI exam we also applied a high-resolution T2 CISS sequence (TR/TE = 5.3/2.4 ms; matrix 266 × 256; FOV 16 × 16 cm; voxel size 0.6 × 0.6 × 0.6 mm), which showed excellent contrast between the CSF and the brain parenchyma, indicating its suitability for precise morphological analysis and estimation of passability through the aqueduct of Sylvius. Also, sagittal and axial cardiac-gated phase-contrast sequences (TR/TE = 24/7.2 ms; matrix 256 × 256; FOV = 22 × 22 cm; voxel size 0.9 × 0.9 × 5 mm) were applied for qualitative assessment of CSF movement through the aqueduct of Sylvius. Volumetric analysis at all MRI exams was performed on T2 coronal slices (TE/TR = 6000/84 ms; matrix = 384 × 384; FOV = 21 × 18, 7 cm; voxel size = 0.5 × 0.5 × 4 mm) using Analyze 8.1 software (Mayo Clinic, Rochester, MN, USA).

**Figure 1:**
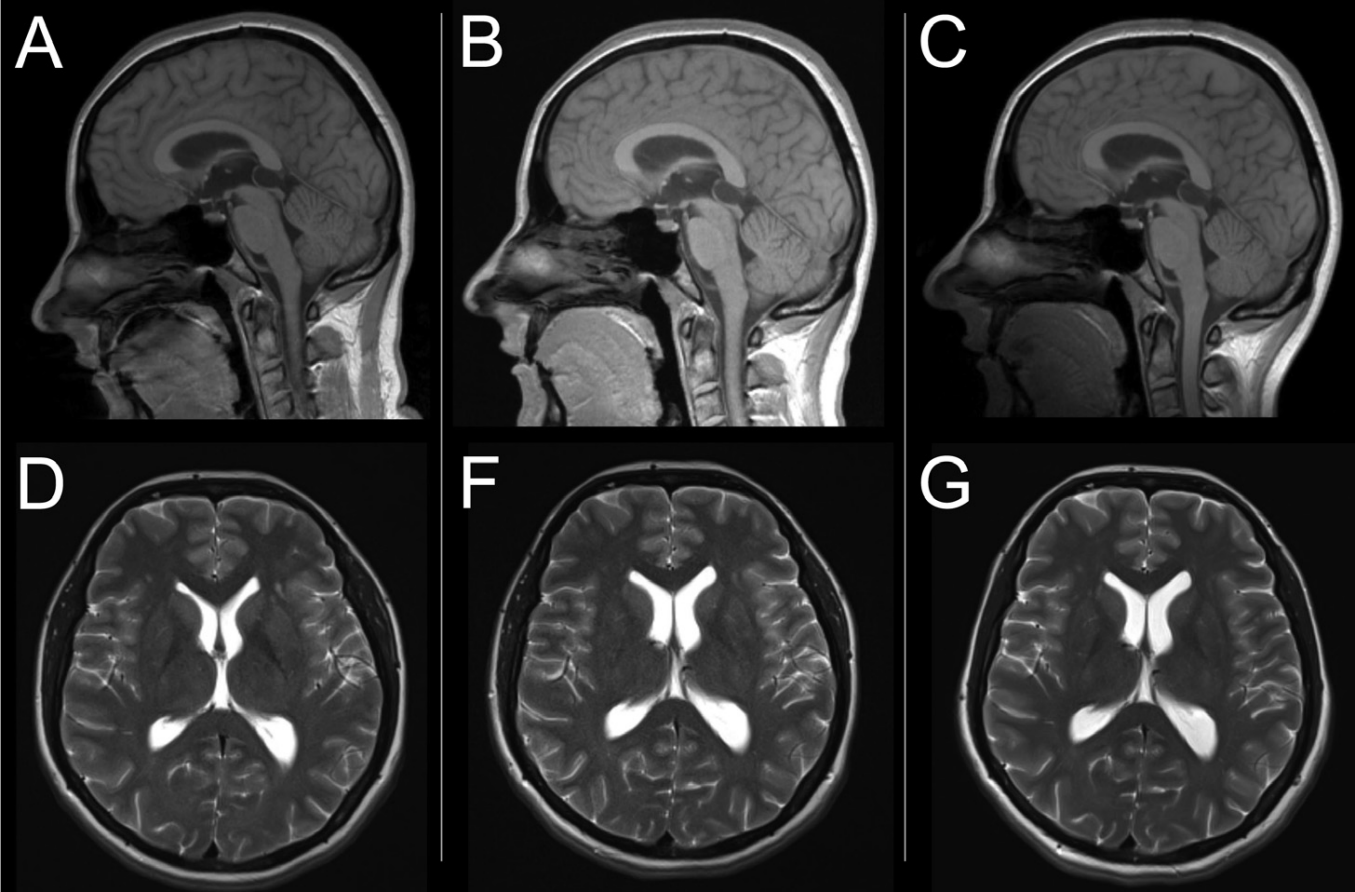
Sagittal T1 slices at the first (**A**), second (**B**), and third (**C**) magnetic resonance imaging (MRI) exam showed a large pineal cyst with compression of quadrigeminal plate and near-obstruction stenosis of the aqueduct of Sylvius. Axial T2 slices at the level of lateral ventricles at the first (**D**), second (**F**), and third (**G**) MRI exam showed a minimal enlargement of ventricular system between the first and second MRI exam.

A series of three MRI examinations showed a large pineal cyst (17 × 10 × 15 mm), which compressed the quadrigeminal plate and caused severe stenosis of the aqueduct of Sylvius (Figure 1 A-C). During the five-year follow up, MRI findings of the pineal cyst and aqueductal stenosis were unaltered. Axial MRI slices at the level of lateral ventricles showed a minimal enlargement of the ventricular system (Figure 1 D-G). No MRI exam showed signs of transependymal CSF transudation, subependymal dissection, or spontaneous ventriculocisternostomy, which according to classic hypothesis could be a compensatory protection against severe hydrocephalus development.

**Figure 2:**
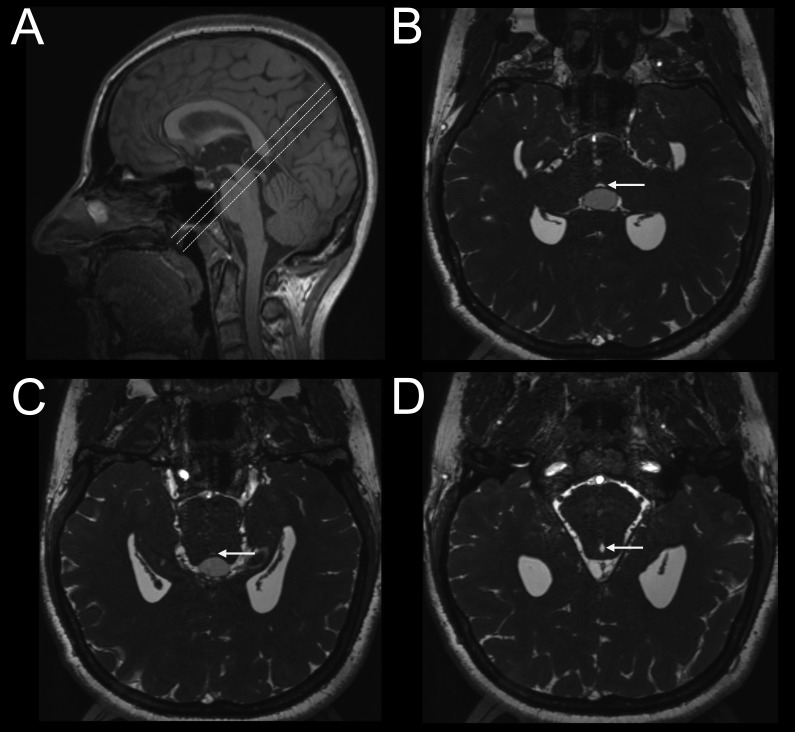
(**A**) Sagittal T1 slice with three dotted lines representing the planes used for magnetic resonance imaging (MRI) analysis of the aqueduct of Sylvius. (**B**) Constructive interference of steady state (CISS) T2 sequence reformatted to the plane of the most cranial dotted line showed compression of quadrigeminal plate with pronounced stenosis of aqueduct of Sylvius. (**C**) CISS T2 sequence reformatted to the plane of the middle dotted line showed near-obstruction stenosis of the aqueduct of Sylvius. (**D**) CISS T2 sequence reformatted to the plane of the most caudal dotted line showed a preserved lumen of the aqueduct of Sylvius in the caudal part of canal.

Volumetric analysis showed a slight increase in the size of the lateral and the third ventricles. At the first MRI exam, summated volume of the lateral and the third ventricles was 42.57 cm^3^, at the second it increased to 47.15 cm^3^_,_ and at the third to 48.73 cm^3^. High-resolution T2 CISS sequence performed at the third MR exam showed a severe stenosis of the aqueduct of Sylvius, which was reduced to almost a virtual diameter, smaller than the size of a single voxel (Figure 2). At the third MR exam, axial and sagittal cardiac-gated phase-contrast sequences were applied for qualitative evaluation of CSF dynamics through the aqueduct of Sylvius. These sequences are sensitive to CSF movement induced by systolic and diastolic cardiac cycles but in our patient no detectable CSF movement through the aqueduct of Sylvius was observed (Supplementary video 1(supplementary video 1) and Supplementary video 2(supplementary video 2)). On other standard sequences there were no artifacts caused by CSF movement, which are usually present in patients with preserved communication between the third and the fourth ventricle. Our structural MRI findings indicated a long lasting near-obstruction stenosis of the aqueduct of Sylvius, while functional cardiac-gated phase-contrast sequences implied a complete blockade of CSF communication between the third and the fourth ventricle.

## Discussion

Despite the presence of a large pineal gland cyst, a near-obstruction stenosis of the aqueduct of Sylvius, and a complete absence of CSF movement through the aqueduct between the third and the fourth ventricle in our patient, there were no signs of hypertensive triventricular hydrocephalus development, as it would be expected according to the classical CSF hypothesis (2,3). We observed a minimal increase in the third and lateral ventricles volume, which could not be explained by CSF accumulation due to the blockade of CSF flow through the aqueduct of Sylvius. According to the classical hypothesis, CSF flow blockade at the aqueduct level would lead to more pronounced structural MRI changes and also to the development of clinical symptoms characteristic for triventricular hypertensive hydrocephalus.

A similar clinical picture has been described in patients with obstructive hydrocephalus following the treatment with Holter ventriculo-cardial shunt (9,10). Namely, 27% of 127 treated children showed so good recovery that the ventriculo-cardial shunt was removed. Some of the patients did not show any symptoms for 1-12 years after shunt removal. After the removal, a CSF system obstruction was still present and CSF “secreted” inside the ventricles could not reach the presumed site of absorption outside of the ventricular system. This condition is known as “arrested hydrocephalus” and it has some similarities with our case.

Similar results were also obtained on experimental animals after a complete artificial obstruction of the aqueduct of Sylvius, which did not induce an enlargement of ventricular system or increase in ventricular CSF pressure (8). In addition, animals with a complete obstruction of the aqueduct of Sylvius did not show any sign of transmantle pressure gradient, which is a prerequisite for an acute hydrocephalus development (9,10). This transmantle pressure gradient in our experiments was induced only after an artificial CSF infusion by pump (imitation of CSF “secretion”) into the ventricular system proximal to the site of the obstruction (8). It should be emphasized that the effects of aqueductal obstruction in experimental animals were monitored only during 2-3 hours, while in the previously described study on children (11,12) and in our patient the follow up lasted for years (Figure 1). Thus, both this case report and our previous experiments on animals show that an obstruction of the aqueduct of Sylvius or severe near-obstruction stenosis (13) do not inevitably lead to a significant enlargement of the ventricular system.

Although these clinical observations cannot be explained by the classic CSF hypothesis, they can be explained by our new hypothesis of CSF physiology, which offers a different perspective on the mechanisms responsible for the hydrocephalus development (5-7). According to this hypothesis, CSF is not actively produced predominantly by the choroid plexuses and does not flow unidirectionally (14,15) to the cortical subarachnoid space, to be passively absorbed through the arachnoidal villi. This means that CSF can be permanently produced and absorbed inside the brain ventricles (16), as well as inside the entire CSF system, as a consequence of water filtration and reabsorption through the capillary walls into the interstitial fluid of the surrounding central nervous system (CNS) tissue. This hypothesis (6,7) is strongly supported by a recent molecular study of Igarashi et al (17), who analyzed water influx into the CSF in aquaporin-1 (AQP-1) and aquaporin-4 (AQP-4) knockout and wild-type mice using a newly developed water molecular MRI technique. They concluded that the water influx into CSF was regulated by AQP-4, known to be responsible for water homeostasis of the pericapillary space, and not by AQP-1 found in the choroid plexuses. Therefore, if filtration and reabsorption of CSF inside isolated ventricles occur through the capillary walls into the interstitial fluid of the surrounding brain tissue, the aqueduct of Sylvius obstruction cannot be the only cause of hydrocephalus development. However, if there are other pathological processes that impair filtration and reabsorption of fluids on the capillary level (eg, bleeding, infection, tumor, toxic substances, etc), accumulation of interstitial fluid and CSF could take place, leading to hydrocephalus development (4). This situation is similar to the previously mentioned experimental condition that was created by an infusion of artificial CSF proximal to the obstruction site, in which a rapid increase in ventricular CSF pressure and transmantle pressure gradient were induced (8). Also this hypothesis can explain the recently reported case of severe acute hydrocephalus development after enteroviral meningitis in a two and half-year-old child with the third ventricle arachnoid cyst (18).

In conclusion, our previous experimental data (8) and the presented case report indicate that an occlusion or blockade of the aqueduct of Sylvius cannot be the single cause of acute hydrocephalus but should rather be considered as a one of the preferential factors for the hydrocephalus development.
